# Dialysis Purification of Integrase-DNA Complexes Provides High-Resolution Atomic Force Microscopy Images: Dimeric Recombinant HIV-1 Integrase Binding and Specific Looping on DNA

**DOI:** 10.1371/journal.pone.0053572

**Published:** 2013-01-14

**Authors:** Tatsuaki Tsuruyama, Tonau Nakai, Rei Ohmori, Munetaka Ozeki, Keiji Tamaki, Kenichi Yoshikawa

**Affiliations:** 1 Department of Anatomical, Forensic Medicine, and Pathological Studies, Graduate School of Medicine, Kyoto University, Kyoto City, Kyoto Prefecture, Japan; 2 Department of Forensic Medicine and Molecular Pathology, Graduate School of Medicine, Kyoto University, Kyoto City, Kyoto Prefecture, Japan; 3 Department of Pathology, Graduate School of Medicine, Kyoto University, Kyoto City, Kyoto Prefecture, Japan; 4 Department of Mechanical and Aerospace Engineering, Graduate School of Engineering, Tottori University, Tottori, Japan; 5 Department of Physics, Graduate School of Science, Kyoto University, Kyoto City, Kyoto Prefecture, Japan; Institute of Human Virology, United States of America

## Abstract

It remains difficult to obtain high-resolution atomic force microscopy images of HIV-1 integrase bound to DNA in a dimeric or tetrameric fashion. We therefore constructed specific target DNAs to assess HIV-1 integrase binding and purified the complex by dialysis prior to analysis. Our resulting atomic force microscopy analyses indicated precise size of binding human immunodeficiency virus type 1 (HIV-1) recombinant integrase in a tetrameric manner, inducing formation of a loop-like or figure-eight-like secondary structure in the target DNA. Our findings regarding the target DNA secondary structure provide new insights into the intermediate states of retroviral integration.

## Introduction

Retroviruses integrate their genome into the genome of host cells in a process catalyzed by the enzyme integrase, which binds the 5′- and 3′- termini of retroviral long terminal repeats (LTRs) and integrates them into the host cell's DNA. Elucidating the underlying mechanism of integration will necessitate that we first obtain a more complete understanding of the transitional states involved in the binding of HIV-1 integrase to target DNA. Hare et al. recently reported a crystal structure of prototype foamy virus integrase complexed with its cognate DNA [1] that is suggestive of a tetrameric structure. HIV-1 integrase forms stable tetramers and associates with the transcriptional coactivator LEDGF/p75, which is an essential cofactor for HIV integration [2], [3]. Inhibitors have been developed to target tetrameric integrases [4], [5]. To date, no convincing visualizations of integrase tetramerization have been reported, perhaps due to difficulties in generating a tetramer stable enough to visualize.

In an effort to visualize integration intermediate states, we previously prepared target DNAs rich in 5′-CA and 5′-TG dinucleotides recognized by HIV-1 integrase [14] and successfully detected the formation of integrase oligomers using atomic force microscopy (AFM). This and other studies provided data that greatly advanced our understanding of the intermediate states of retroviral integration [6], [7], [8]. The results of recent studies of the binding of nucleoproteins to retroviral cDNA ends suggest that integrase forms oligomers at the ends of the retroviral genome [9], [10].

In the present study, we used a purified recombinant integrase produced in Sf9 insect cells for formation of the integrase-DNA complex. Kotova et al. estimated that the number of integrase molecules that bind to individual strands varies significantly [9], suggesting that evaluations based on the number of molecules binding to target DNA are more error prone than evaluations based upon measurement of the volume of DNA-integrase complexes. In studies involving AFM observations, background noise often impedes accurately measuring the intensity of the objective peaks. In the present study we therefore employed dialysis to reduce the background noise sufficiently to resolve the oligomeric structure of DNA-integrase complexes using AFM. Our study also revealed that oligomeric integrase digests the target DNA at the oligomeric integrase-binding site with secondary structure formation. Our AFM data indicated that recombinant HIV-1 integrase forms a tetrameric structure in complex with target DNA. These results will enhance current understanding of the molecular mechanism of retroviral integration.

## Materials and Methods

### Incubation of integrase with target DNA

Recombinant HIV-1 integrase was kindly provided by Dr. Tomokazu Yoshinaga, Shionogi Institute for Medical Science, Japan, who previously described the HIV-1 integration signal sequence [11]. This integrase was prepared free of nucleases. The target DNA consisted of approximately 4.0 kb of *x_k_y_k_* : (5′-GTGGAGGG*CAGT*-3′) _k_(5′-*ACTG*GGAAGGGAC-3′)_k_ or *x′_k_y′_k_* : (5′-GAGGAGGGTAGC -3′)_k_(5′-*GCTA*CCCTCCTC-3′)_k_, where 160<*k*<170 and the italicized portions of the sequence (*CAGT* and *ACTG*) represent sequence that originated from the LTR end of the HIV-1 provirus. In *x′_k_y′_k_*, *CAGT* and *ACTG* have been replaced. A 10-µL volume of solution containing 10 ng of target DNA was combined with 50 ng of recombinant integrase in 10 µL of binding buffer and incubated for 0 to 20 min at 30°C to prevent excess digestion by endonuclease activity that would occur during incubation at 37°C. The binding buffer was comprised of 25 mM MnCl_2_, 80 mM potassium glutamate, 10 mM mercaptoethanol, 10% DMSO, and 35 mM MOPS (pH 7.2).

### Dialysis of the integrase-DNA complex

The integrase-DNA complex was dialyzed in order to reduce the background noise and eliminate artificial aggregation to obtain convincing images. Following incubation, 1.0 mL of PBS was added to the integrase-target DNA solution and the mixture was then gently injected into a dialysis bag (Slide-A-Lyzer Dialysis Cassettes, 20K MWCO, Pierce Biotechnology, Rockford, IL) and dialyzed against PBS for 48 h at 4°C in order to remove the MOPS and Mn^2+^. The PBS was exchanged six times every 2 hour. Finally, the dialysis product was gently withdrawn from the bag and analyzed using AFM or digested with *Sca*I (New England Biolabs, Ipswich, MA). This digestion was performed in order to prevent the formation of complicated DNA structures resulting from intersecting of the target DNA or supercoiled structure. We frequently observed such structures, which made it difficult to distinguish the monomeric integrase or artifacts in AFM observations due to heterogeneous fixation on the background mica.

### Atomic force microscopy

A droplet of a solution containing target DNA and 10 mM spermidine was then placed on a freshly cleaved piece of mica (30–50 mm). Spermidine was used to aid the adsorption of DNA molecules onto the mica surface. After 5 min, the adsorbed DNA was washed with water and dried with N_2_ gas. The DNA molecules were analyzed in air at room temperature using an NVB100 atomic force microscope (Olympus Optical Co., Ltd., Tokyo, Japan; AFM controller and software: Nanoscope IIIa, Digital Instruments, Veeco, Camerillo, CA) operated in the tapping mode [12]. The integrase volume was calculated by
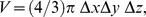
where *Δx*, *Δy*, and *Δz* denote the sizes of the three-dimensional axis. Details are described in the Results section below. These molecular size values were determined using *Jmol*, an open-source Java viewer for chemical structures in 3D (http://www.jmol.org/) on the basis of 3D X-ray structure analysis.

### Electropherogram

Electrophoresis was performed following incubation of 10 ng of target DNA with 50 ng of integrase for various periods ranging from 0 to 20 min. The resulting signal intensities were transformed using Multigauge software (Fujifilm, Tokyo, Japan).

### Statistical analysis

Statistical significance was assessed by unpaired *t*-tests using SPSS software (SPSS, Chicago, IL). *P*-values <0.01 were considered statistically significant.

## Results

### Binding of integrase to an artificial repeat sequence

**Figure 1:**
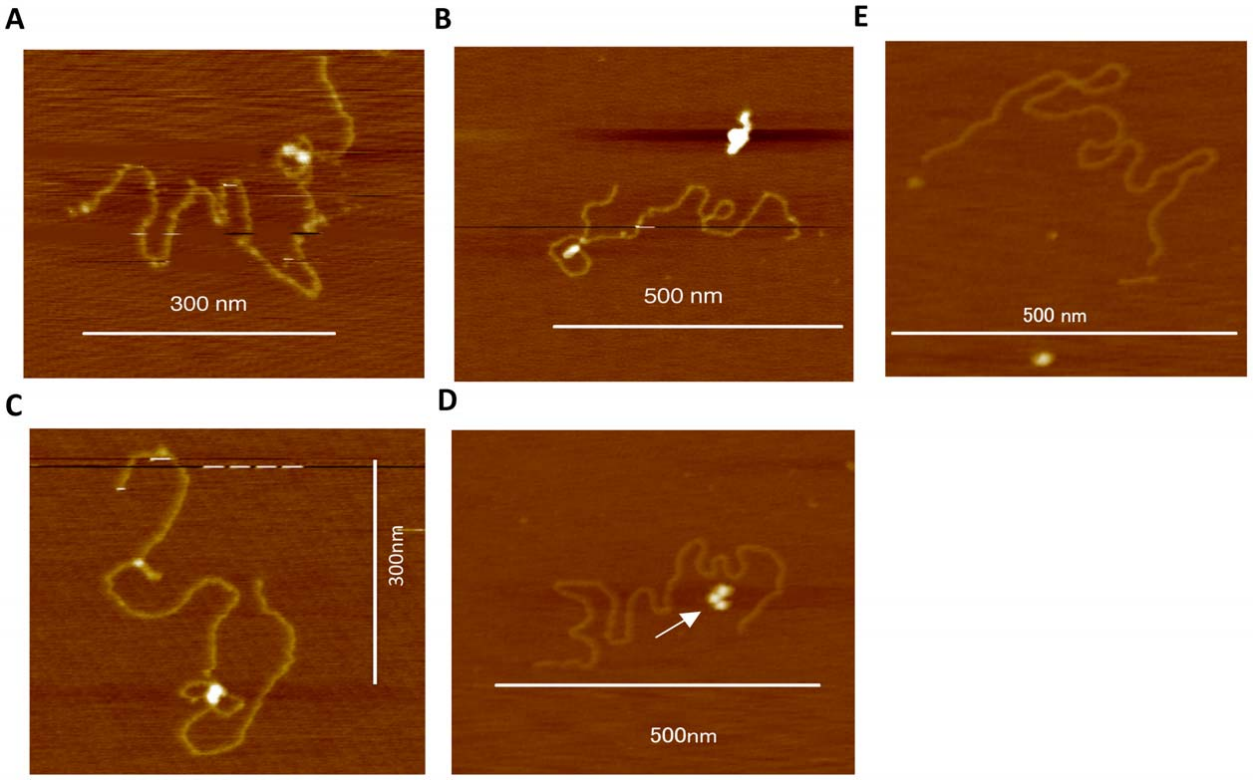
AFM observation of integrase-target DNA complexes. (A, B) Target DNA *x_k_y_k_* bound to HIV-1 integrase forming a loop-like structure or (C) a figure-eight-like structure. (D) DNA showing no HIV-1 integrase binding, where the arrow indicates unbound integrase. (E) Control experiment using *x′_k_y′_k_* including a *Sca*I digestion site AGTGACT. Bars indicate scale.

**Figure 2:**
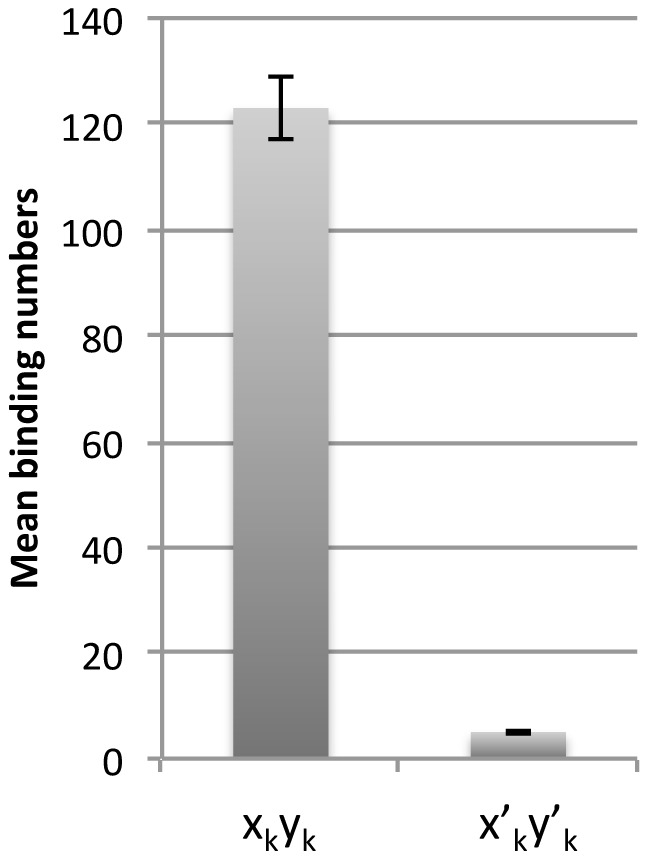
Number of integrase molecules bound in target DNA and modified DNA complexes. Mean number of oligomeric integrases binding to the target DNA or modified target DNA strands (per one hundred strands) (mean ± s.d.). Results were determined from 5 independent experiments.

*x_k_ y_k_* or *x′_k_ y′_k_* was incubated with recombinant HIV-1 integrase in a reaction buffer containing 40 mM MnCl_2_. Samples containing integrase and target DNA were dialyzed prior to analysis in order to obtain high-resolution images. Without this purification, high background was observed. After dialysis, AFM analysis indicated that HIV-1 recombinant integrase bound to the target DNA *x_k_y_k_*. The target DNA formed a *twin-like* globular structure. Well-defined loop-like (Figures 1A, 1B) or figure-eight-like structures (Figure 1C) were observed around the oligomeric integrase. The presence of free monomeric and oligomeric integrase indicated that integrase does not bind to target DNA incubated prior to dialysis in the absence of MnCl_2_ (Figure 1D). Binding of oligomeric integrase to *x′_k_y′_k_* was less observed in the presence of MnCl_2_ than *x_k_y_k_* (Figure 1E, See figure 2).

### Modification of target DNA significantly represses integrase binding

The target DNA *x′_k_y′_k_* was also incubated with recombinant HIV-1 integrase in a reaction buffer containing 40 mM MnCl_2_. Subsequent AFM analysis indicated that significantly fewer HIV-1 recombinant integrase molecules bound to the *x′_k_y′_k_* target DNA compared to the *x_k_y_k_* target DNA (5 molecules bound to *x′_k_y′_k_* strands vs. 123 molecules bound to *x_k_y_k_* strands, *P*<0.01) (Figure 2). These data indicate that the focal nucleotide sequence has a significant influence on integrase binding.

### Integrase binds to target DNA in a tetrameric fashion

**Figure 3:**
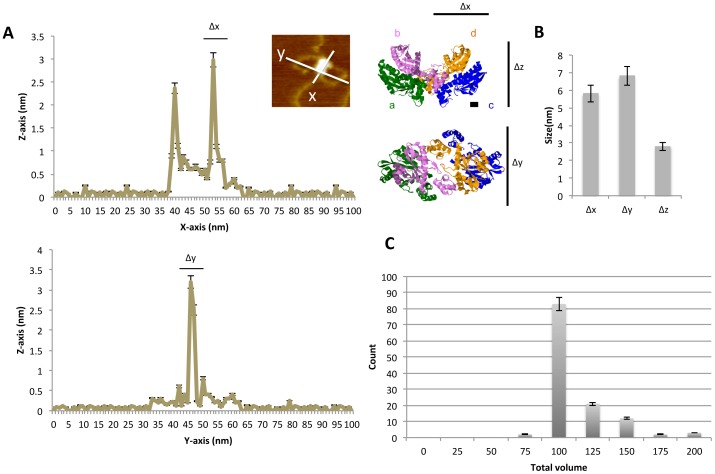
Distribution of the size of integrase-target DNA complexes. (A) Line graphs showing representative measurement of the diameter across a twin-like structure of integrase bound to the DNA strands along the x- and y-axes. The upper and lower graphs show the longer and shorter diameter of the twin-globule structure individually. The photo in the inset is identical to the photo shown in Figure 1C. To the right are 3D crystal structures of integrase obtained from X-ray data (PDB 1K6Y) [15]. Four integrase molecules associate in a tetrameric fashion in the DNA binding form. Monomers are represented by (a) green, (b) purple, (c) blue, and (d) orange. The diameter of the integrase dimer along the y-axis is approximately 5 nm, measured by the distance between two amino acids (E69 of c and I191 of d), represented in red. The thickness along the z-axis is approximately 3 nm, measured by the distance between Q62 of c and C65 of d, represented in red. These values were determined using *Jmol*. The scale bars are equivalent to approximately 1 nm. (B) Size of tetrameric integrase along the x-, y-, and z-axes (n = 123). (C) Histogram showing the distribution of the estimated volume (nm^3^) of the twin-like integrase structures bound to target DNA strands (n = 123).

The objective molecular size was evaluated based on peak intensity using an AFM controller and software. Representative trace data are shown in Figure 3A [15], [16]. According to the protein database (PDB), the size of the x-, y-, and z-axes of the objective twin-like structure should be about Δx≈5, Δy≈5, and Δz≈5 nm. The diameter corresponds to the long axis of the monomeric integrase structure obtained from X-ray data [15], [16]. Our data revealed mean sizes of 5.8±0.3, 6.1±0.1, and 3.2±0.2 nm for Δx, Δy, and Δz, respectively (Figure 3A, B). Taking into account that fixation of the protein on the mica surface reduced the thickness to 60% of its actual value, the thickness (Δz) of the oligomeric integrase applied to the surface of mica corresponds to 3.2 nm/0.6 = 5.3 nm, implying that one integrase molecule was piled atop another [17], [18], [19]. The present Δx and Δy data indicate that the majority of the complexes contained dimeric integrase. The mean volume of the oligomeric integrase was 112±12 nm^3^ assuming an elliptical structure (twin-like structures; n = 123 DNA strands; Figure 3C). The volume distribution corresponds to a molecular mass of between 62 and 65 kDa [9]. Because the mass of monomeric integrase is 32 kDa, our data indicate that the majority of the complexes contained dimeric integrase. Therefore, the twin-like structure definitely binds to the target DNA in quadruplets.

### Integrase binding generates target DNA secondary structures

**Figure 4:**
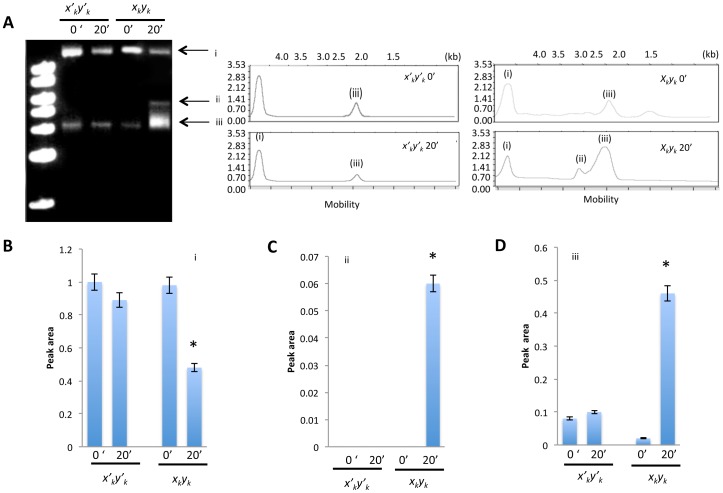
Electrophoretic analysis of circular DNA bound to integrase. (A) Electrophoretic analysis of control and target DNA following incubation with integrase. Graphs show electropherograms of signals corresponding to bands (i)–(iii). Peak area of individual signals (B) (i), (C)(ii), and (D)(iii) was determined using Multigauge software. Control lane depicts the electrophoresis of the random-sequence control DNA.

To evaluate the effect of integrase binding on the target DNA structure, electrophoretic analysis was performed directly without digestion of the circular *x_k_y_k_* or *x′_k_y′_k_* DNA following the integrase binding reaction. In addition to the target DNA (i) and presumably supercoiled signal (iii), an upper signal (ii) and an evident mobility fluctuation of (iii) were observed (Figure 4A). This analysis showed that the relative areas associated with electrophoretic signals (ii) and (iii) were significantly higher in *x_k_y_k_* relative to *x′_k_y′_k_* (Figures 4B–D, *P<0.01), confirming that integrase binding to *x_k_y_k_* induces fluctuation in the DNA structure.

## Discussion

The results of the present study, which utilized target sequences originating from repeats of the HIV-1 cDNA ends, provide clear evidence that recombinant integrase forms a tetrameric complex on target DNA. A tetramer model of the HIV-1 integrase-DNA complex was previously constructed in an effort to predict the integrase residues that interact with the LTR termini [3], [13]. A repeat unit of target DNA was identical to the DNA for use in an *in vitro* integration assay in our previous study [14]. The target DNA was expected to maintain the integrase binding during the dialysis process. Sequence motif *CAGT* in the target DNA was observed in the end of HIV-1 ling terminal repeat, which was essential to HIV-1 integration [14], [20]. Indeed, this *CAGT* is a part of integration signal sequence that was reported by Yoshinaga et al [11]. In addition to this DNA sequence feature, the target DNA yielded a fluctuating secondary structure in the reaction buffer as shown in Figure 4, and this fluctuation contributes to the integration efficiency, probably because of the tension created by the secondary structure.

The present data clearly indicates that the twin-like structure binds to the target DNA in quadruplets, i.e., in a tetrameric fashion, which is consistent with the results of previous crosslinking experiments [9]. Our data also indicate that integrase induces a significant bend or fold in the target DNA, thereby producing a characteristic secondary structure. When the DNA was maintained in a circular structure the pattern of integrase-DNA binding was more complicated and it was frequently difficult to observe the binding complex. Therefore, we digested the complex in order to produce a more distinct secondary structure that would be easier to observe. By releasing the topological tension associated with the circular structure of the target DNA, we were able to reliably measure the volume of oligomeric integrase and thereby determine that the enzyme forms a tetrameric complex with the target. We found that dialyzing the integrase-DNA solution significantly reduced the background noise resulting from aggregation induced by MnCl_2_, thereby improving the resolution of subsequent images. This method enabled us to obtain high-resolution images of the structure of the integrase-DNA complex from which we were able to determine that dimeric integrase binds tightly to the target DNA in a symmetric fashion, producing a stable tetrameric configuration. Although our study is not applicable to *in vivo* integration studies, the data do provide new insights into how HIV-1 integrase interacts with host cell target DNA.
